# Empirical Overview of Benchmark Datasets for Geomagnetic Field-Based Indoor Positioning

**DOI:** 10.3390/s21103533

**Published:** 2021-05-19

**Authors:** Imran Ashraf, Sadia Din, Soojung Hur, Gunzung Kim, Yongwan Park

**Affiliations:** Department of Information and Communication Engineering, Yeungnam University, Gyeongbuk, Gyeongsan-si 38541, Korea; imranashraf@ynu.ac.kr (I.A.); sadiadin@yu.ac.kr (S.D.); sjheo@ynu.ac.kr (S.H.); gzkim@yu.ac.kr (G.K.)

**Keywords:** magnetic field data benchmarks, indoor positioning, smartphone sensors, benchmark analysis, magnetic field positioning

## Abstract

Indoor positioning and localization have been regarded as some of the most widely researched areas during the last decade. The wide proliferation of smartphones and the availability of fast-speed internet have initiated several location-based services. Concerning the importance of precise location information, many sensors are embedded into modern smartphones. Besides Wi-Fi positioning, a rich variety of technologies have been introduced or adopted for indoor positioning such as ultrawideband, infrared, radio frequency identification, Bluetooth beacons, pedestrian dead reckoning, and magnetic field, etc. However, special emphasis is put on infrastructureless approaches like Wi-Fi and magnetic field-based positioning, as they do not require additional infrastructure. Magnetic field positioning is an attractive solution for indoors; yet lack of public benchmarks and selection of suitable benchmarks are among the big challenges. While several benchmarks have been introduced over time, the selection criteria of a benchmark are not properly defined, which leads to positioning results that lack generalization. This study aims at analyzing various public benchmarks for magnetic field positioning and highlights their pros and cons for evaluation positioning algorithms. The concept of DUST (device, user, space, time) and DOWTS (dynamicity, orientation, walk, trajectory, and sensor fusion) is introduced which divides the characteristics of the magnetic field dataset into basic and advanced groups and discusses the publicly available datasets accordingly.

## 1. Introduction

Indoor positioning and localization have been regarded as important research areas during the last decade. In 2020, the projected number of smartphone users reached approximately 3.5 billion, indicating a 9.3% increase from 2019 [1]. The wide proliferation of smartphone and internet usage paved the way for a large number of online services which are collectively called location-based services (LBS). To offer precise location information for LBS using smartphones, a rich variety of sensors are embedded in modern smartphones like accelerometer, gyroscope, magnetometer, barometer, Wi-Fi, lux meter, and Bluetooth, etc. Besides providing the necessary information for various user-oriented operations like optimal display, phone orientation change, and user-specific profile management, these sensors’ generated data can be used to categorize user movements, estimate user’s location and track user’s walking trajectory, etc. Smartphones are used to provide location information both outdoor and indoor, of course, the use of positioning technology varies. For example, for outdoor positioning global positioning system (GPS) meets the positioning requirement [2]. Conversely, the indoor environment poses several physical barriers for GPS to work properly such as roofs, walls, and other similar interference sources, signal absorption, and reflection, etc. Consequently, various positioning technologies have been specifically devised or implemented to provide accurate and robust location information for indoor environments.

Human spent a large portion of their time indoors and 80% to 90% of our time involve indoor activities at places like offices, train stations, shopping malls, and university, etc. which makes indoor positioning a potential research area [3,4]. Concerning the importance of indoor positioning for rescue and emergency services as declared by E911 calls, several positioning technologies have been introduced over the years such as like Wi-Fi [5,6], radio frequency identification (RFID) [7], infrared [8], ultra-wideband [9], Bluetooth [10], and geomagnetic field-based positioning [11], etc. Despite the availability of several above-mentioned technologies, infrastructureless approaches have been focused on their working principle that does not involve additional infrastructure. Besides Wi-Fi, the geomagnetic field has been widely investigated for indoor positioning over the last few years.

Using the geomagnetic field data (referred to as magnetic field for simplicity in the rest of the paper) has several advantages over other approaches. First of all, it uses the subsisting infrastructure like the Wi-Fi and (commercial-off-the-shelf) applications to estimate the current position of the user. However, the Wi-Fi positioning has several limitations. For example, intrinsic limitations of radio wave propagation cause a substantial change in the received signal strength (RSS) which degrades the performance of Wi-Fi-based fingerprinting solutions [12]. Additionally, signal absorption, multipath shadowing, and shading during dynamic environments cause signal fluctuation and affect positioning accuracy. Human mobility, human body loss, and diversity in antenna and hardware also affect the RSS value [13]. Secondly, unlike the Wi-Fi, UWB, Bluetooth, IR, and RFID-based positioning, the magnetic field-based positioning does not require access points (APs), beacons, structured lights, and tags for locating a user indoors. The magnetic field data is a natural phenomenon and its value can be measure both indoors and outdoors using only a magnetometer which is present in smartphones. Thirdly, the magnetic field data has shown long-term stability and been reported in several works [14,15]. Its behavior is more stable and less affected than Wi-Fi, Bluetooth, and RFID technologies, etc. Similarly, the mutation of the magnetic field data is minimal than radio frequency-based approaches which increase its suitability in dynamic environments.

Despite the advantageous properties of magnetic field-based indoor positioning, it has several challenges that require attention. First, changes in indoor infrastructure involving ferromagnetic materials like steel, iron, nickel, etc., and paramagnetic materials like aluminum cause a substantial change in the magnetic field intensity leading to large positioning error. Secondly, the complex behavior of the user during the positioning process such as call listening, phone in the pockets (front or back), texting, and navigation, etc. changes the magnetic field intensity and affects the positioning accuracy if device orientation is not tracked properly. It also makes it very difficult to model all possible orientations of the smartphone as such activities change from user to user. Similarly, although not substantial, the height of the user affects the magnetic field data. Thirdly, the diversity of the smartphone is one of the big challenges for magnetic field-based indoor positioning. Various smartphone companies use magnetic sensors from different vendors. These sensors show different intensities concerning their accuracy, noise tolerance, and specificity, and sensitivity. Even various brands of the same smartphone tend to show variation in the magnetic field data. Additionally, the embedded magnetic sensor may lose configuration in the proximity of ferromagnetic materials like large speakers and show very different magnetic field data if not reconfigured. Last but the most important is the benchmark dataset used for evaluating the positioning algorithm. The magnetic field-based positioning is in its infancy and lacks good benchmarks. Often, the training and testing data are collected by individual researchers and are not made public. The lack of proper and public benchmark leads to positioning results specific to the data used in a given environment and produces very different results when used elsewhere. This study discusses the magnetic field data benchmarks that are used for positioning. In brief, this study makes the following contributions

- Important characteristics of a benchmark are spotlighted concerning the magnetic field data. Such characteristics are divided into two groups DUST (device, user, space, and time diversity) and DOWTS (dynamicity, orientation, walking speed, the trajectory of the path, and sensor fusion) and highlighted for their importance to perform positioning in the indoor environment.
- The importance and necessity of each element of DUST and DOWTS are discussed by visualizing the magnetic field data collected during extensive experiments over a longer period. Furthermore, the influence of DUST and DOWTS is analyzed concerning indoor positioning accuracy with the magnetic field data.
- A brief description of the magnetic field data benchmark is given where various aspects of the benchmark are elaborated. These benchmarks are analyzed in the light of drawn elements of the magnetic field benchmark.
- A comprehensive overview of limitations, potential of the benchmark datasets is given and the prospects of a good benchmark dataset for evaluating the magnetic field-based indoor positioning approaches are outlined.

The current study focuses on the description of the available magnetic field data benchmarks and is organized in the following manner. Several important research works on geomagnetic field data are discussed in Section 2. Section 3 points out important characteristics required for a magnetic field benchmark. A brief overview of the benchmarks is described in Section 4 which analyzes their importance concerning the defined elements of the magnetic field data benchmarks. Section 5 contains the discussion of the prospects for a good magnetic field data benchmark and how it can be achieved. In the end, the conclusion is given in Section 6.

## 2. Related Work

Due to the ubiquity, simplicity of implementation, and availability of embedded magnetic sensors in smartphones, a large body of research works can be found in the literature that uses magnetic field data. The magnetic field-based indoor positioning approaches can be categorized as a magnetic field only positioning and hybrid approaches. The former employs the magnetic field data alone and conventionally a fingerprinting approach is used for the positioning. The latter on the other hand utilizes the data from multiple smartphone sensors, as well as, different technologies such as Wi-Fi, vision, Bluetooth, and PDR, etc.

Initial research works on the magnetic field data focus on the magnetic field data alone and validate the use of indoor magnetic field anomalies for positioning. The research [16], for example, investigates the use of magnetic field data alone for indoor positioning and shows that it provides low positioning accuracy. Furthermore, the more the number of magnetic field elements, the higher the positioning accuracy. However, to ensure high accuracy, the data from other technologies is advised. Similarly, the research [17] studies the influence of walking speed on the positioning performance of the magnetic field data and uses the dynamic time warping for matching the magnetic signatures of various lengths.

Several approaches utilize magnetic field data from multiple smartphone sensors and multiple technologies to refine the positioning accuracy. For example, For example, the authors adopt a multi-sensor approach in [15] for indoor positioning and use inertial measurement unit (IMU) sensor data like the gyroscope, accelerometer along with the magnetic field and Wi-Fi data to provide refined location information of the user. Similarly, the use of vision along with the machine and deep learning approaches tend to show superior results than the conventional fingerprinting approaches. The authors provide a multi-sensor approach in [18] that utilizes smartphone camera images for initial scene identification which is later used to restrict the search space for the magnetic field database. Opportunistic Wi-Fi data are also used to correct the position periodically. Along the same lines, a hybrid approach is used for multi-floor indoor positioning with a smartphone camera, magnetic field, and data from IMU sensors in [19]. Later, the use of smartphone heterogeneity is investigated in a few research works. For example, research works [14,20] analyze the positioning performance of magnetic field data using different smartphones. Different positioning accuracy is reported for different smartphones suggesting that the influence of smartphone heterogeneity can not be ignored.

The use of the machine and deep learning approaches are made in several research works that utilize magnetic field data to train and later predict the user’s position. For example, [11] uses an ensemble classifier to predict the user’s current position. Several convolutional neural networks (CNNs) are trained on a set of features extracted from the magnetic field data. The output from each CNN is used to make the final prediction through an ensemble approach which incorporates the IMU data to find the best solution. Similarly, the use of magnetic field patterns is investigated in [21] where the CNNs are trained on the image of the magnetic field data patterns. Each of the magnetic field components is combined into an image which serves as the fingerprint for each location. Results suggest high accuracy and low smartphone dependency for this approach. Recently, an augmented magnetic field vector is used in [22] where the augmented magnetic field data are produced for different locations. Experiments show that using the augmented magnetic field vector minimizes the drift error and increases the positioning accuracy.

The above-discussed research works investigate different aspects of the magnetic field data concerning indoor positioning accuracy. However, the experiments are not extensive and the data collected or used during the experimentation are not provided.

## 3. Important Elements of Magnetic Field Benchmark Dataset

**Figure 1:**
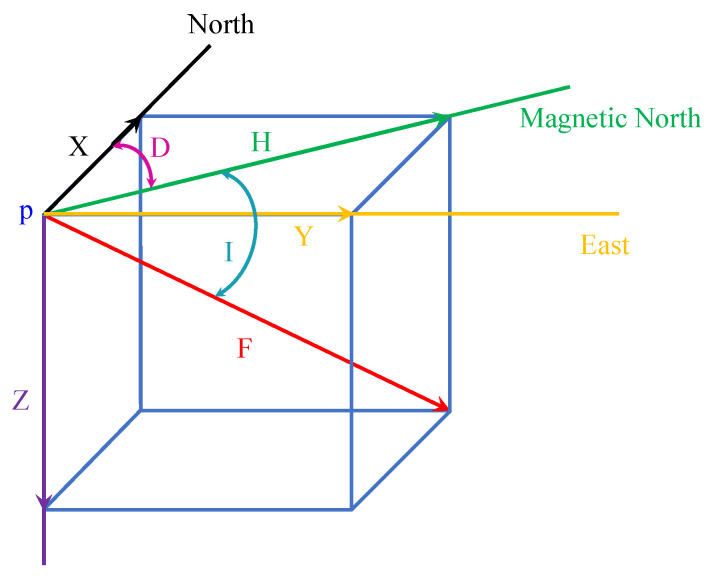
The components of the magnetic field at a given point *p* on earths surface.

Two types of applications are found for using the magnetic field data for indoor positioning: artificially generated magnetic field and the natural magnetic field is also known as the earth’s magnetic field. This study focuses on the benchmarks used for the latter category. The earth’s magnetic field is a natural phenomenon caused by the convection currents in the outer layer of the earth. For a given point *p* at the earth’s surface, the magnetic field vector can be represented by *x*, *y* and *z* components or *F*, *D* and *I* where *x*, *Y* and *z* denotes the north, east and downward elements of the magnetic field while *D* and *I* are declination and inclination angles and *F* represents the total magnetic field intensity. These components are shown in Figure 1 and calculated using the following formulas

(1) $$\begin{array}{c} F=\sqrt{X^{2}+Y^{2}+Z^{2}} \end{array}$$

(2) $$\begin{array}{c} D=arctan\frac{Y}{Z} \end{array}$$

(3) $$\begin{array}{c} I=arctan\frac{Z}{H} \end{array}$$

where *H* given in Equation (3) is the horizontal magnetic field intensity and calculated using

(4) $$H=\sqrt{X^{2}+Y^{2}}$$

The point *p* is assumed to be at the center of a Cartesian coordinate system. For further details on the magnetic field components and related formulas, the readers are referred to [23,24].

The intensity of the magnetic field does not change abruptly in a natural outdoor environment within a small area. However, man-made constructions involving ferromagnetic materials interfere with the natural distribution of the magnetic field and cause disturbances also called anomalies as they introduce errors in direction sensing. However, such anomalies are reported to show the unique spatial distribution for indoor environments and used as fingerprints for indoor positioning by several works [16,17,25]. While the magnetic field data is pervasive, inexpensive, and does not require additional sensors or hardware for positioning, it has several inherent challenges that require extensive research. For this purpose, the benchmarks used for evaluating the magnetic field-based positioning approaches should have several important characteristics. For this purpose, eight important features are spotlighted for the magnetic field benchmark which is essential to evaluate a positioning approach to generalize its results. These characteristics are categorized under two groups called primary characteristics and advanced characteristics. Primary characteristics are termed DUST (device, user, space, and time diversity) while the advanced characteristics are named DOWTS (dynamicity, orientation, walking speed, trajectory, and sensor fusion).

### 3.1. Device Diversity

**Figure 2:**
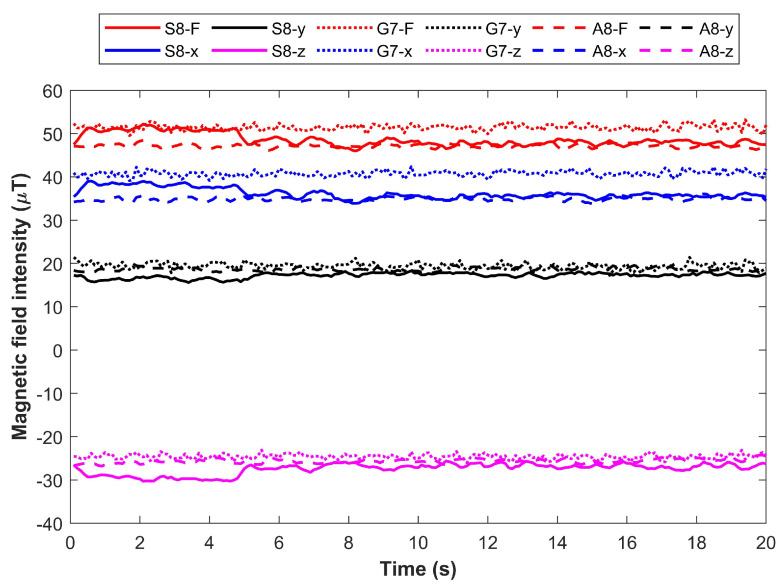
Magnetic field data from three different smartphones. Variation in the data can be seen for different devices. The variation may increase or decrease for other smartphones.

Predominantly, smartphones are used for magnetic field-based indoor positioning, and the diversity of the magnetometer used in various smartphones substantially changes the performance of the positioning approach. Several research works report the change in the magnetic field intensity when heterogeneous devices are used for data collection [15,26,27]. The difference in the measured intensity may be attributed to one or more of these factors. First, smartphone embedded magnetometers are based on microelectromechanical systems (MEMS) which are tiny but inexpensive sensors and data precision is not their primary attribute. Such sensors are famous for their small size to be used in smartphones and cost only a few dollars. Secondly, various smartphone companies like Samsung, Apple, Nokia, and Huawei, etc. use the magnetometer from various vendors and their data collection procedure, as well as, the hardware may vary. Even various brands of the same smartphone company use various magnetometer concerning their version, price, and size, etc. [27,28]. Such diversity makes it necessary to test the positioning approach from the data collected using various companies’ smartphones, as well as, various brands of the same smartphone company. For illustration, Figure 2 shows the magnetic field data collected at the same location using three different smartphones such as Samsung Galaxy S8, LG G7, and Samsung Galaxy A8. The smartphones have different embedded magnetometers which result in different measured values for the magnetic field data. Samsung Galaxy S8 has Asahi Kasei AK09916C, Samsung Galaxy A8 has Asahi Kasei AK09918C, and LG G7 has Asahi Kasei AK09915C. While the change in the measured values is comparable, the difference is visible for different smartphones.

**Figure 3:**
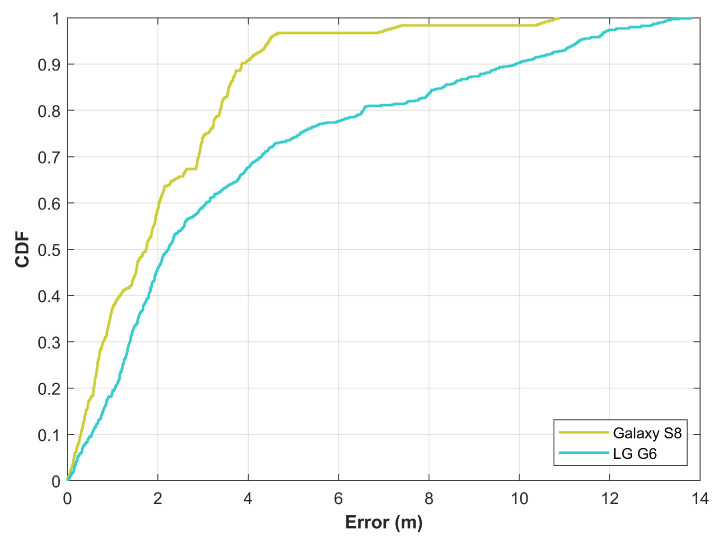
The CDF graph for magnetic field data based positioning using two different smartphones.

To show the importance of data collection from multiple smartphones, experiments are performed for the magnetic field data. For this purpose, a state-of-the-art indoor positioning approach is implemented [14] using two different smartphones including Samsung Galaxy S8 and LG G6. Several runs are performed to calculate the position while the user walks along a dedicated path in the indoor using the navigation mode. Positioning results are shown in Figure 3.

Results indicate that the influence of device heterogeneity can not be ignored. The performance of the Galaxy S8 is superior to that of the LG G6. Of many reasons for the performance difference, the two most probable reasons are the changing magnetic field data from different smartphones and the choice of a smartphone to make the fingerprint database. Equipped with different magnetometers, different smartphones show different values for the measured data which affects the positioning results even when the same positioning algorithm is used. Secondly, it is not possible to make separate databases for each smartphone, and conventionally only a single database is built using one of the smartphones which are later used with all the smartphones. For current experiments, the database is made using the Galaxy S8 while positioning is carried using both smartphones. Consequently, the performance of the positioning algorithm is affected. Results prove that the data collection from multiple smartphones is of significant importance when making a magnetic field benchmark dataset.

### 3.2. User Diversity

**Figure 4:**
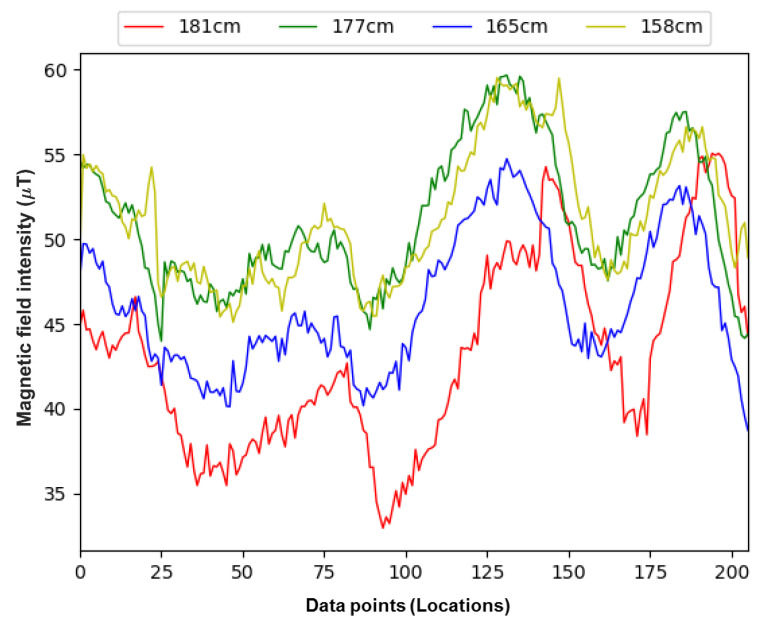
Impact of the users’ heights on the magnetic field data. The data collection scenario is the same where the data is collected during the same hour of the day. The *x*-axis shows the data points at different locations when the user is walking along the path.

Different users hold the smartphone at different heights that vary the magnetic field data intensity even different users collect the data at the same location with similar smartphone holding position, as reported in [21,29]. The variations in the collected magnetic field data are caused by the height of the user and the altitude of the held smartphone as altitude tends to change the intensity of the magnetic field data [30]. Similarly, the device holding style of users may vary leading to changes in the data and different positioning accuracy even when the same positioning approach is used. Figure 4 shows the data involving two male and two female surveyors of different heights. It can be observed that both the magnetic field data intensity, as well as, the sequence of magnetic patterns change for different users. From Figure 4, we can see that the difference in the measured values from different users is higher than what is observed in Figure 2. There are two reasons for this difference: location change and user change. The data in Figure 2 are measured at the same location for 20 s and portray the influence of various smartphones. On the other hand, Figure 4 shows the data collected on different locations while walking. Consequently, the change may be different at different locations. Secondly, different users collect the data for Figure 4 and the difference in the data may be the user’s height and change due to the smartphone magnetometer.

**Figure 5:**
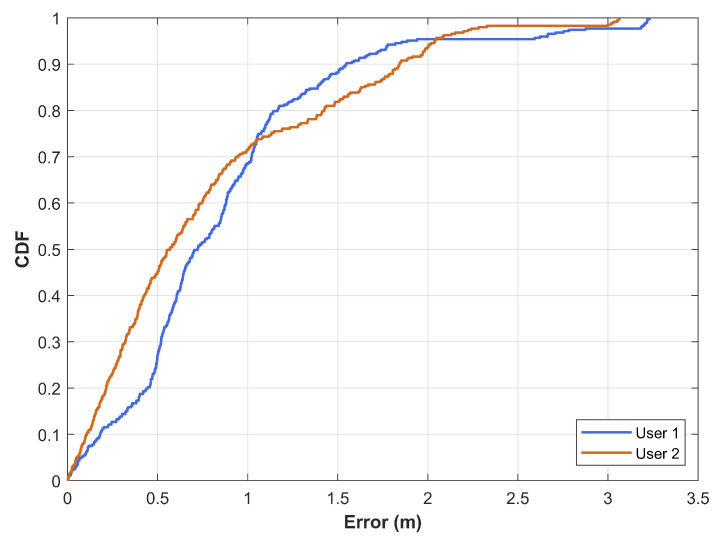
The CDF graph for positioning results involving two users with different heights. The heights of the users are 165 cm and 171 cm for user 1 and user 2, respectively.

While the magnetic field data varies concerning different users, its influence on the positioning performance is not studied very well. Experiments are performed to study the influence of different users on the positioning performance of magnetic field data. Two users participated in the experiments for this purpose using the same positioning algorithm. Samsung Galaxy S8 is used in an indoor environment to conduct experiments. Figure 5 shows the positioning performance with users of different heights. Results show that the influence of users’ height is minimal for the magnetic field data as the positioning performance is marginally different for two users. However, the performance is almost similar for the two users because the mean error for users 1 and 2 are 0.8599 m and 0.7964 m, respectively. Similarly, the values for median error and standard deviation are also very close.

### 3.3. Space Diversity

For the most part, magnetic field-based positioning utilizes the fingerprinting approaches where fingerprints are collected at specific marked locations during the offline phase or training phase. Determining the suitability of a fingerprint is of significant importance as it directly affects the positioning accuracy. The two most important characteristics of a fingerprint are spatial differentiation and temporal stability [31,32]. Spatial differentiation refers to the notion that at various locations in an indoor environment the magnetic field data is unique to identify that location. However, it is not entirely true for magnetic field data as similar intensity may appear at several locations. Space diversity for the magnetic field data refers to that the magnetic field benchmark should contain the data collected in different buildings. Since the indoor setting/infrastructure of a building interferes differently with the magnetic field, the distribution of the magnetic field data may be very different for different buildings. Besides, this distribution may vary for the construction material used for the building, as reported in [28] that buildings of old materials or stones may show a smooth magnetic field with no disturbances.

A good magnetic field benchmark should contain the data for various building types, as well as, geographically well-separated buildings. Besides, the magnetic field data is used for floor identification, so the data from multiple floors of the same building would hold extra benefit for floor identification problem [33]. Analogous to the Wi-Fi positioning that has different positioning errors for different size indoor places, the magnetic field positioning varies concerning the indoor space. To corroborate this hypothesis, several experiments are conducted in three different buildings of a university campus. The buildings are geographically well separated from each other and have different indoor settings and available areas for positioning. The dimensions of three buildings are 90×32 m${}^{2}$, 88×32 m${}^{2}$, and 50×35 m${}^{2}$ for building 1, 2 and 3, respectively.

**Figure 6:**
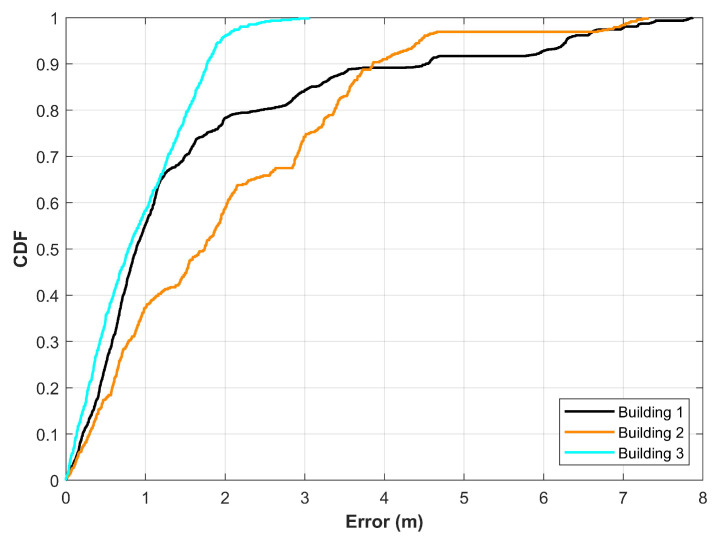
Results for magnetic field-based positioning in three buildings with different indoor environment and dimensions.

Figure 6 shows the positioning results for three buildings. It can be seen that the positioning performance is different for different buildings. It may be argued that the performance is affected due to different path geometries used during the experiments. It is a strong point and can not be ignored completely. However, during the experiments, we found that due to the indoor environment, these buildings have different distributions of the magnetic field data where building 3 has more unique fingerprints than buildings 1 and 2. Similarly, a higher number of similar fingerprints are found for different locations which are well separated for building 2. Consequently, the positioning performance for building 2 is poor than other buildings. For example, the mean error for buildings 1, and 2 is 1.5909 m, 1.9802 m, respectively, while building 3 has a mean error of 0.9047 m only. Keeping in view the results presented here, the data from multiple buildings are highly desirable to analyze the performance of the magnetic field data-based indoor positioning.

### 3.4. Time Diversity

As previously pointed out that temporal stability is one of the two important characteristics of a good fingerprint. Several research works suggest that the magnetic field data exhibit temporal stable signatures to be used as location fingerprints [17,34]. Time diversity characteristics of a benchmark imply that the data should be collected over a longer period of time during different intervals so that the performance of the positioning approach can be extensively evaluated [35].

It is also important to study the long-term behavior of the magnetic field data as it is reported to mutate over time [15,20]. The world magnetic model (WMM) is modified every five years to incorporate the magnetic field data mutations [36]. While the mutation of the magnetic field data is very low than Wi-Fi signals, several studies report the mutation of the magnetic field data over time [20,37]. Such mutations represent the natural change in the magnetic field intensity, given there is no infrastructure change in the indoor environment involving ferromagnetic materials.

**Figure 7:**
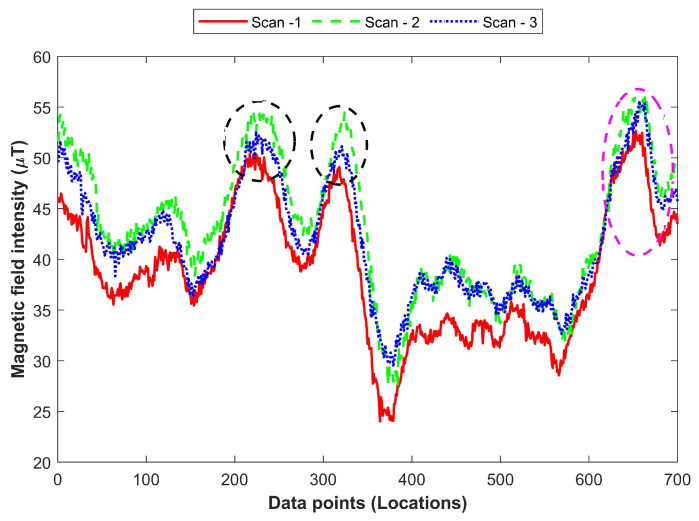
Time related mutation of the magnetic field data. The circled portions of the figure highlight the points with abnormal changes.

The data shown in Figure 7 have been collected over 6 to 8 months using the same smartphone, i.e., Samsung Galaxy S8. As displayed in the figure, the intensity of the magnetic field data varies over time. The change in the magnetic field data may be abrupt at certain points as indicated in black circles in Figure 7. Similarly, changes in the magnetic field data may be attributed to the varying speed of the user. For example, the magenta circled are shows the data where the change in the data is due to the walking speed of the user. Besides the influence of time, the effect of a smartphone embedded magnetometer can not be ignored completely as it tends to show slightly different data when collected over different times even using the same smartphone.

### 3.5. Dynamic Behavior of Magnetic Field for Temporary Indoor Changes

**Figure 8:**
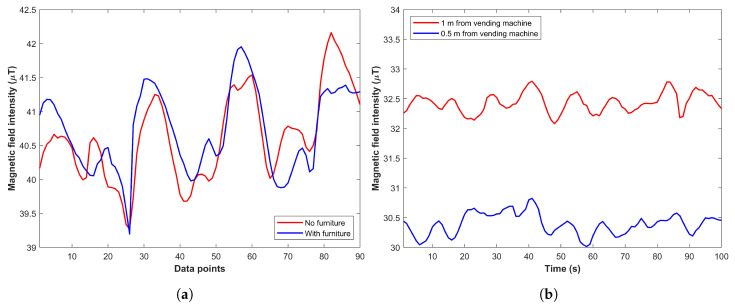
The impact of ferromagnetic materials’ proximity, (**a**) Influence of furniture, and (**b**) Vending machine [28].

One important factor for the benchmark dataset is the inclusion of the data showing dynamic changes. Such dynamic changes can occur by temporary infrastructure changes indoors such as the presence of a refrigerator, industrial fans, furniture, or a vending machine as pointed out in [28,38]. Despite that several works have regarded the magnetic anomalies as stable over time, the data collected in the presence of various items alongside the data collection path helps to evaluate its impact on the localization algorithms [39,40,41]. The influence of such changes depends on the type of materials placed indoors and their mass. For example, wood cupboards and furniture do not affect the magnetic field data. On the contrary, placing the ferromagnetic materials would greatly interfere with the magnetic field data and affect the positioning performance. For example, the impact of placing a chair with steel legs and a vending machine is shown in Figure 8.

### 3.6. Orientations of Device

**Figure 9:**
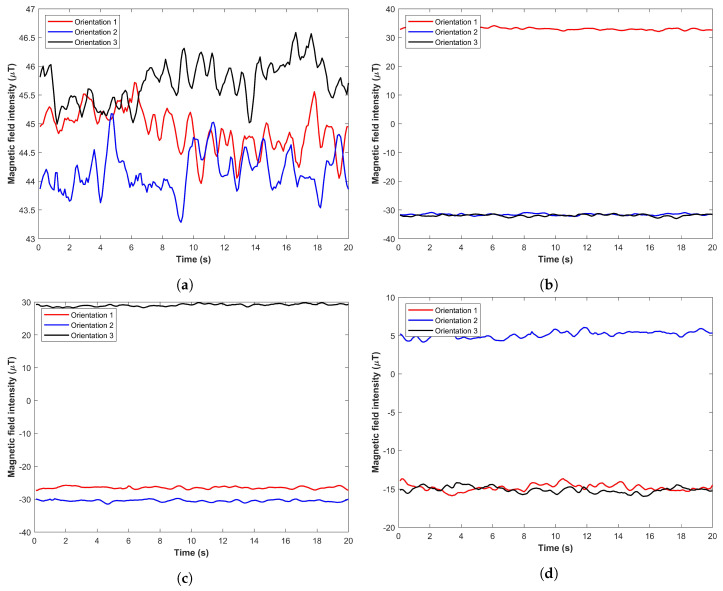
The magnetic field data for three orientations as described, (**a**) Magnetic *F*, (**b**) Magnetic *x*, (**c**) Magnetic *y*, and (**d**) Magnetic *z*.

Despite the large number of positioning approaches focusing on the magnetic field data, only a few investigated the impact of device orientation on positioning accuracy. Predominantly, the magnetic *x*, *y*, and *z* are used to make fingerprints, and change in smartphone orientation substantially changes the value of these elements [24]. So the common assumption for the majority of the magnetic field-based positioning approaches is to work with a fixed orientation where the user can walk in either direction but can not change orientation [42,43]. The user walks in various directions with a fixed orientation, traditionally, the smartphone is put in his hand called navigation mode. The data collected using a smartphone magnetometer contains noise, the user changing the orientation of the smartphone requires the transformation of the data to global coordinates which introduces both complexity and error. Additionally, it is not possible to model all the possible actions of the user while walking, such as call listening, texting, phone in the pocket (front or back), swinging in the hand and hold in right or left hand, etc. However, the benchmarks should contain the data of most common orientations at least such as navigation, call listening and phone swinging to analyze the impact on the positioning accuracy to model the real-life situations. Various orientations substantially affect the magnetic field data and even the sub-orientations for one category of orientation cause a huge change in the data. For example, the phone in the front pocket of pants is one orientation but the phone in the front pocket may have three possible orientations: upside down with LCD facing the user, upside down with LCD facing outward, and downside up with LCD facing the user. Figure 9 shows the magnetic field data for the above-mentioned three scenarios and represents the change in the magnetic field.

Smartphone orientation influences the positioning accuracy of a magnetic field-based approach substantially. Predominantly, the navigation mode is used for evaluating the performance of the magnetic field data-based positioning, and the user does not change the orientation of the smartphone during the test. However, change in the smartphone orientation is possible for the user but it requires the transformation of the data. The magnetic field fingerprint database is made using the earth coordinate system. The readings from the smartphone need to be transformed into an earth coordinate system for localization. Let $M={\left[m_{x},m_{y},m_{z}\right]}^{T}$ be the magnetic field vector, $M_{s}$ be magnetic field readings at smartphone coordinate system, $M_{e}$ be the data at the earth coordinate system, the relationship between the smartphone and earth coordinate system can be defined as [44,45]

(5) $$M_{s}=R_{x}\left(\phi \right)R_{y}\left(\theta \right)R_{z}\left(\psi \right)M_{e}$$

where ψ, θ and ϕ represents the yaw, pitch and roll, respectively, while $R_{x}\left(\phi \right)$, $R_{y}\left(\theta \right)$, and $R_{z}\left(\psi \right)$ show their corresponding rotation matrices.

**Figure 10:**
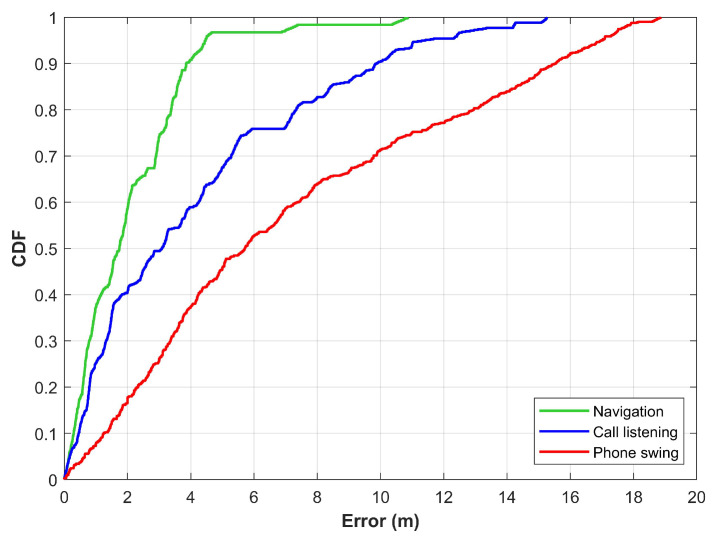
The CDF graphs for three orientations of the smartphone including ‘nvaigation’, ‘call listening’, and ‘phone swinging’.

Due to the noise in the readings, the rotation matrices are not exactly correct which affects the transformation process as well. Consequently, the positioning results vary significantly while using different smartphone orientations. For further verification, three smartphone orientations are used to conduct experiments such as ‘navigation’, ‘call listening and ‘phone swinging’. During the call listening mode, the user places the smartphone by his right ear in an upward position making an angle of approximately 45${}^{\circ }$ while the phone swinging mode includes moving the smartphone in the right hand while walking. Results for the above-mentioned three orientations are given in Figure 10. Results suggest that the positioning performance is slightly degraded when switching from navigation to call listening while substantially reduced when swinging the smartphone during the positioning process. Consequently, the magnetic field data from multiple smartphone orientations are required for magnetic field benchmark datasets.

### 3.7. Walking Speed Variation

Conversely to the Wi-Fi data which is slightly affected by the walking speed of the user, the magnetic field data has a substantial impact when the users walk at various speeds [28,46]. While the sequence or the pattern formed by the magnetic field data remains similar, its length varies with the walking speed of the user, and matching the pattern with the pre-built database leads to singularity problem [47]. Often, dynamic time warping (DTW) is used to resolve the issue where two sequences of different lengths are compared. However, it adds computational complexity when matching longer sequences of the data. Despite that, the data with a varying walking speed of the user is required in the benchmark to analyze the performance of the positioning approach.

**Figure 11:**
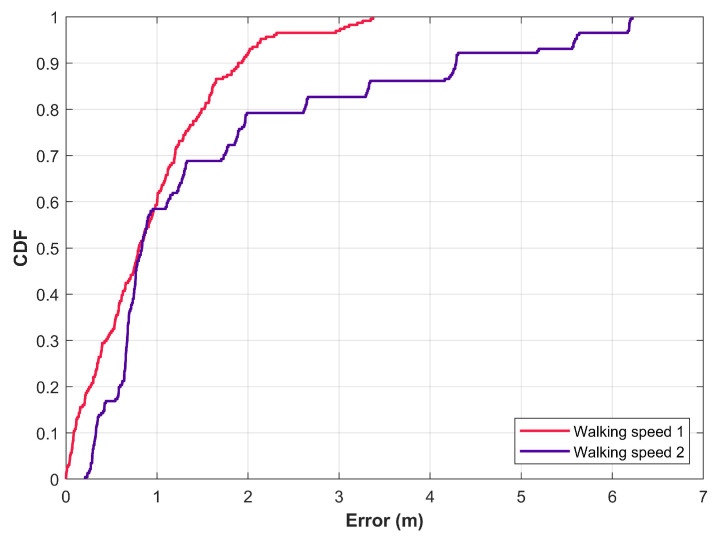
Performance of magnetic field-based indoor positioning approach involving various walking speeds.

The users walking speed, as well as, the walking style varies from one user to another. So, the influence of users’ walking speed can be analyzed when the magnetic field data benchmarks provide the data for various walking speeds. Walking speed influences the positioning performance of magnetic field-based positioning approaches. To analyze this impact, two users participated in several experiments in an indoor building using Galaxy S8. The walking speed is measured using the accelerometer data of the smartphone. Positioning results are given in Figure 11 where the walking speed 1 and 2 are 0.98 m/s and 1.43 m/s, respectively. Results show that the performance of the positioning approach is affected when the walking speed is increased. During fast walking, smartphone shaking may be higher due to the hand movement leading to noise in the measured data which ultimately affects the positioning performance. At a slower speed, the user movement is consistent and data are smooth than at a fast speed. So, the data collected at different walking speeds have different positioning performances. So, for evaluating the performance of the magnetic field-based positioning approaches, the benchmark datasets should provide the data from the users walking at different speeds.

### 3.8. Trajectory of Path Used for Experiments

The trajectory of the path used for experiments is an important characteristic that needs to be considered for the magnetic field benchmarks. Initial works on using the magnetic field data followed simple path trajectories for feasibility studies for both handheld devices and robot navigation [16,32,48]. Magnetic field-based positioning show higher accuracy with simple path trajectories, however, for deployment in real-life scenarios, experimentation with complex path trajectories is required. Complex path trajectories include turns in various directions, as well as, the data from various indoor set up such as large spaces, rooms, and corridors, etc. Complex path trajectories involving turns and frequently varying directions show reduced accuracy with magnetic field-based positioning [15,20,47,49].

**Figure 12:**
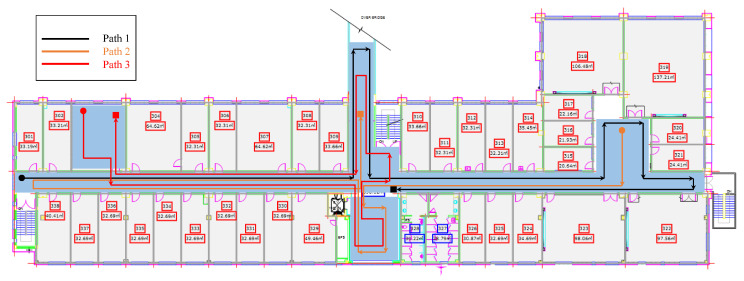
Path geometry used to analyze the impact of path complexity on magnetic field-based indoor positioning [14].

**Figure 13:**
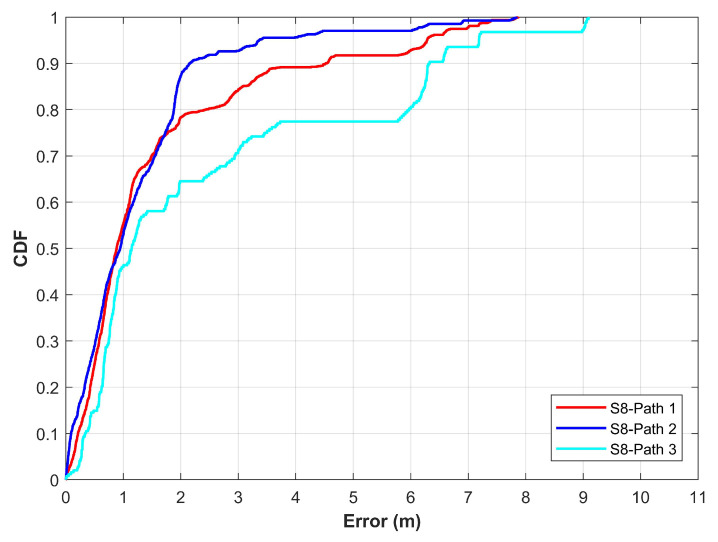
The CDF graphs for various path geometries used to evaluate the performance of magnetic field-based indoor positioning [14].

Generally, it would appear that path geometry has no impact on the positioning performance of the magnetic field data, however, it is not completely true. It is possible that multiple simple paths may have the same positioning performance but the same is not true between a simple and complex path geometry. Complex paths tend to affect the positioning performance of the magnetic field-based positioning approach. To analyze the need for the magnetic field data from multiple path geometries, experiments are performed using three paths with different levels of complexity. The paths used for the experiments using Galaxy S8 are shown in Figure 12. Path 1 is simple as compared to both path 2 and path 3. Path 3 is more complex than path 1 and path 2 and involves several turns at short places. Experimental results are shown in Figure 13. Results show that the positioning results vary concerning the path geometry used for positioning. Even though the same positioning approach is used with a single smartphone for the same indoor place, the positioning performance is different for different paths. Hence, the data from different path geometry can be very useful to analyze the positioning performance of state-of-the-art magnetic field-based indoor positioning approaches.

### 3.9. Sensor Fusion

The positioning accuracy of the magnetic field-based approaches is limited when the magnetic field data is used alone as the same fingerprints may exist at several distant locations in large buildings or big halls. For this reason, the data from multiple sensors are combined to enhance the positioning accuracy [19]. For the most part, the data from the inertial measurement unit (IMU) of the smartphone are used with the magnetic field data. The smartphone IMU contains several sensors such as an accelerometer, gyroscope, and barometer, etc. which helps to increase the positioning accuracy. In addition, using Wi-Fi or Bluetooth data helps to narrow down the search space in the magnetic field database and increases the accuracy [18]. For sensor fusion, the benchmark should contain the data from several sensors at each location [50]. Additionally, data from the accelerometer and gyroscope contains noise which leads to uncertainty and error in the position called drift. The drift is accumulated as the user continues walking and needs to be recorrected. Periodic position updates using complementing technology such as Bluetooth and Wi-Fi helps to resolve this issue.

## 4. Overview of Magnetic Field Benchmark Datasets

Wide research interest for the magnetic field data for indoor positioning led to the introduction of several datasets that are used as benchmarks in many research papers. Despite that, the number of available benchmarks is still few and lacks important characteristics of a good benchmark. This study includes all the datasets that have been used for indoor positioning using the magnetic field data and discusses them concerning the outlined characteristics of DUST-DOWTS for the magnetic field. The magnetic field benchmark datasets have been utilized in a plethora of indoor positioning approaches including traditional fingerprinting and statistical approaches. Kalman filter, extended Kalman filter, particle filter have been implemented for enhanced position accuracy on these datasets. Similarly, statistical approaches, Bayesian models, and Markov chain models are also reported.

### 4.1. Barsocchi et al.

**Figure 14:**
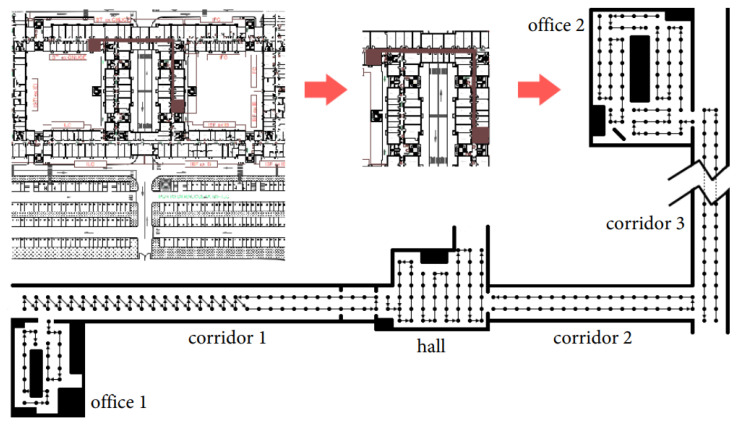
Map and trajectory of the path used for the data collection in [51].

This research focuses on the data collection for Wi-Fi and magnetic field using the same location points [51]. The dataset is publicly available at http://archive.ics.uci.edu/ml/datasets/UJIIndoorLoc-Mag and accessed on 3 May 2021. The data are recorded using a smartphone, as well as, the smartwatch taken at the same time from a human surveyor. The smartphone used for the data collection is Sony Xperia M2 while LG W110G Watch R is used as the smartwatch. The dataset is collected over a single place which contains offices, corridors, and connected corridors through turns. Besides the magnetic field data, the IMU data are collected including accelerometers and orientation sensors. Figure 14 shows the path trajectory that is used for the data collection.

The path trajectory is complex containing several turns and the area is large but several limitations are associated with the dataset. The data is collected over a short time and the impact of magnetic field mutation can not be tested using the dataset. Besides, although the data collected using the smartwatch is important to analyze the change in the magnetic field data concerning user’s movement, only a single smartphone is used and device dependence can not be investigated. Space diversity, i.e., data at various geographically separated buildings, is not used either. The advantage is that the Wi-Fi and IMU sensors data can be used for sensor fusion.

### 4.2. MagPIE

**Figure 15:**
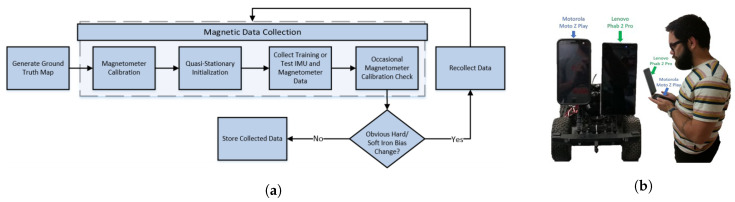
Data collection procedure for MagPIE, (**a**) The pipeline for the data collection, and (**b**) wheeled platform and handheld device for the data collection [38].

MagPIE is a magnetic field dataset introduced in an international conference on indoor positioning and indoor navigation (IPIN) in 2017 [38]. The dataset is publicly available at http://bretl.csl.illinois.edu/magpie and accessed on 3 May 2021. It records magnetic anomalies in the indoor environment using the smartphone IMU sensors. The data are collected in multiple buildings having different dimensions and indoor infrastructure. Data collection is performed using two platforms: handheld smartphones and wheeled robot-mounted smartphones. Motorola Moto Z Play and Lenovo Phab 2 Pro are used for data collection. Ground truth measurements are calculated using Google’s Tange API on Lenovo smartphone. Figure 15 shows the pipeline and data collection platform used for MagPIE.

Despite involving two smartphones and multiple buildings for data collection, MagPIE lacks several characteristics. First, although MagPIE contains the data collected by putting several items beside the path, the long-term behavior of the magnetic field data is overlooked. The path trajectory used to collect the data is not explained as well. In addition, various orientations of the smartphone are not considered for data collection and only one orientation is used with both the robot and handheld device. Furthermore, only a single user collected the data with on walking speed, and the impact of change in user heights and walking speed can not be investigated using the MagPIE.

### 4.3. Torres-Sospedra et al.

**Figure 16:**
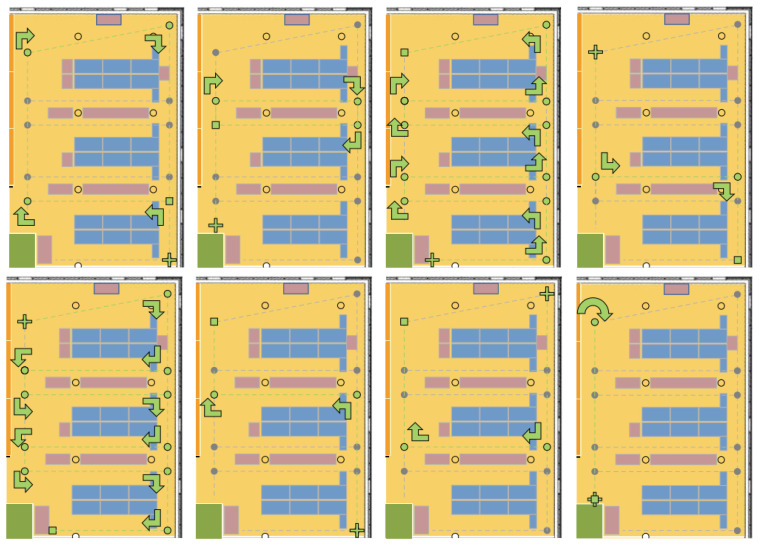
Various paths and trajectories adopted for the data collection in [52].

A dataset is presented in [52] where the Wi-Fi and the magnetic field data are collected within the same indoor environment for evaluation of the magnetic field-based positioning systems. The dataset also contains the IMU sensors data including the accelerometer and orientation sensors. The collection area comprises corridors and intersections and data are collected following two different directions. Data collecting smartphone is Google Nexus 4 with Android 5.0.1 operating system. The dataset is publicly available at http://indoorloc.uji.es/ and accessed on 3 May 2021. Figure 16 shows the path trajectories used for data collection in [52].

While the data are collected from April to September 2014, it does not cover the time required to test the time diversity of the magnetic field data. Similarly, eight corridors and 19 intersections are used for the data collection but they all reside in the same building. The walking speed of the user remains almost the same where the impact of the varying walking speed is analyzed through the resampling using the interpolation. Only a single smartphone with a fixed orientation is used for the data collection.

### 4.4. Miskolc IIS Hybrid IPS

**Figure 17:**
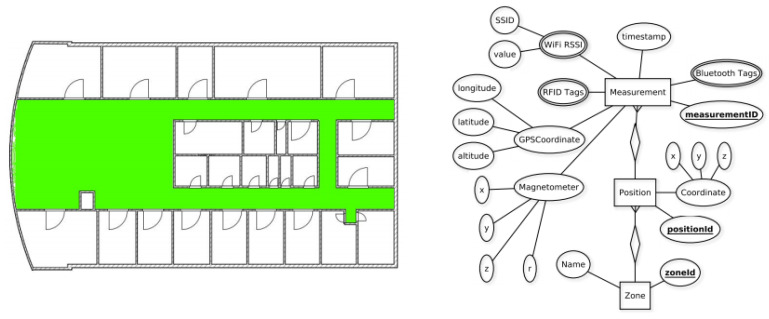
The area for the data collection and the schema of the database. Greed color shows the covered area for the data collection [53].

The dataset aims at providing the data from several technologies to perform hybrid indoor positioning [53]. For this purpose, the data are collected for the Wi-Fi, RFID, Bluetooth, and magnetic field using Samsung Galaxy Young GT-S5360 with Android 4.4.4. However, the unavailability of RFID support on the client device results in an empty record for RFID. A total of 30 Wi-Fi APs and 9 Bluetooth devices. Corridors and halls comprising an area of 465.75 m${}^{2}$ are used for the data collection. ILONA system is used for the data collection which is a client-server and contains the methods, storage, and other business logic on the server-side. The client-side collects the data and sends it to the server for processing and storage. Manually calculated location points are used for the data collection. The dataset is publicly available at https://archive.ics.uci.edu/ml/datasets/Miskolc+IIS+Hybrid+IPS and accessed on 3 May 2021. The area used for the data collection and the schema of the database for Miskolc IIS hybrid dataset are shown in Figure 17.

The area for the data collection is large and wide with the multi-level collection, however, the dataset does not contain the data from multiple buildings. The path trajectory is provided for the dataset and the dataset does not cover the influence of the dynamic behavior. Similarly, user diversity and device diversity are not used for the dataset. The walking speed of the user is almost similar and the data for the user’s varying speeds are not gathered. Moreover, the data are not collected over a longer period of time and spans only a couple of weeks.

### 4.5. UJIIndoorLoc-Mag

**Figure 18:**
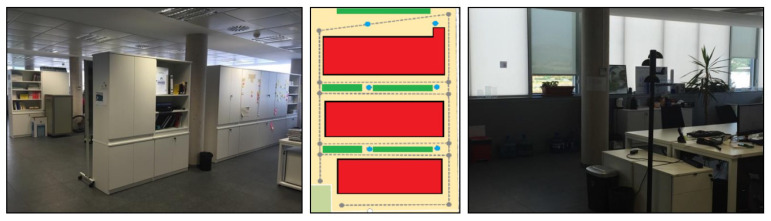
Data collection environment for UJIIndoorLoc-Mag. The Center figure shows the path trajectory used for the data collection. The red area shows the desktop computer tables while the dotted lines indicate the data collection path. Circles indicate the starting and ending points for each data collection path [54].

UJIIndoorLoc-Mag is the magnetic field data which was presented at IPIN 2015 by [54]. The dataset is publicly available at https://archive.ics.uci.edu/ml/datasets/UJIIndoorLoc-Mag and accessed on 3 May 2021. The dataset is collected in a laboratory environment as shown in Figure 18 and the data are collected as continuous samples. The laboratory consists of 8 corridors and 260 m${}^{2}$ space, separated by bookcases and desktop computers. Space is divided into several smaller units for the data collection with a starting and ending spot and the data are collected when the user walks between these points at each 0.1 s. The data also stores the starting and ending position for each path, as well as, the coordinates of path turns in case of corridor intersections. The data are collected separately for training and testing where the test paths are claimed to be complex involving multiple turns in the intersections. Moreover, two different smartphones are used for test data like Google Nexus 4 and LG G3 both running on Android 5.0.

Since the user walking speed during the data collection varies, the IMU data are gathered along with the magnetic field data to perform resampling. The data are gathered over a short period of time within one laboratory environment and the impact of space and time diversity can not be analyzed using the UJIIndoorLoc-Mag dataset. Multiple users collect the data, yet the height information of the data surveyors is not made available. There are no obstacles along the path used for the data collection and users can walk freely without any interruption. The orientation of the smartphone is kept constant, i.e., navigation for the data collection, and the impact of various orientations is not reported.

### 4.6. XJTLUIndoorLoc

A fingerprint dataset to perform indoor localization and trajectory estimation is presented in [55]. The dataset is collected in a multi-story building of Xi’an Jiatong-Liverpool University, Suzhou, China. The dataset contains the data for the magnetic field and Wi-Fi. For data collection, an Android application is developed where the starting location point and smartphone can be entered. The data collection is performed in corridors of 30×3 m${}^{2}$ at different floors of the building while the resolution of the magnetic field data is 60 cm. Single orientation of the smartphone is used for the data collection, however, different postures are employed including normal navigation, phone tilting left and phone placed in an upward direction vertically facing the data collector. Similarly, the data collection is carried out facing the smartphone in different directions at each data collection point. Two smartphones are used for data collection such as Huawei P9 and MiX2 that collect the magnetic field data and Wi-Fi data from 515 APs.

The space structure used for the data collection in [55] is small and the path geometry simple. Despite the data collection from various directions at each location point, a single orientation is used. Similarly, a single user is involved in the data collection for magnetic field and Wi-Fi data. Data are collected for a short period and time-related mutation of the magnetic field data can not be investigated. Moreover, only the magnetic field and Wi-Fi data are available and IMU sensors data are not recorded which helps to track users’ walking direction and attitude. While the data are collected from multiple floors, it does not involve a single building and spatial diversity is not available for the XJTLUIndoorLoc dataset.

The benchmark dataset discussed in this section provides the magnetic field data for indoor positioning. The data can be used for indoor positioning for both parametric approaches, as well as, machine and deep learning techniques, as discussed in Section 2. Providing the ground truth for each dataset instance, the data can be used to train the machine and deep learning approaches with important features from these datasets.

## 5. Discussions and Future Directions

Magnetic field-based indoor positioning and localization is a research area that holds great potential for the future market. With the wide proliferation of smartphones and the availability of the fast internet, today a broad spectrum of applications provide customer services that rely on the user’s position. In addition, emergency response services require precise location information for rescue tasks. Due to the demand from these services, the importance of an accurate position is elevated. Consequently, a broad range of positioning technologies and approaches have been introduced and adopted for positioning, both indoors and outdoors. Indoor environments pose more challenges concerning indoor complex structures and dynamic activities. Also, human mobility in crowded indoor environments poses an extra challenge that severely affects the performance of radio signal propagation especially. As a result, the performance of the Wi-Fi, Bluetooth, and UWB based indoor positioning is crippled in the dynamic environments found at high mobility places like universities, shopping malls, train stations, and airports, etc.

To cope with such challenges, magnetic field-based positioning has emerged as a prospective candidate recently. In addition to being ubiquitous, and easy to implement, it does not require additional installment of APs, tags or beacons, etc. to perform the indoor positioning. The embedding magnetic sensor in modern smartphones can be used to collect the magnetic field anomalies and perform the indoor positioning without additional infrastructure. However, the lack of publicly available benchmark datasets makes it very difficult to compare the performance of the state-of-the-art positioning approaches that use magnetic field anomalies. Over the past few years, several datasets have been presented but they lack several important aspects that are necessary to test a magnetic field-based positioning approach.

First of all, the major problem for magnetic field positioning approaches is to overcome the data variation from magnetic sensors that are embedded in smartphones. A large number of companies with different brands and models makes it very difficult to devise an approach that works seamlessly with all smartphones. However, an approach should be tested with more than one smartphone company like Samsung, LG, Huawei, etc. Android phones cover the major market of smartphones, so the data for android smartphones should be available. Among the five publicly available datasets, only two provide the data from more than one (i.e., two) devices. However, the data from two smartphones are not enough to validate that a particular approach shows similar performance with various smartphones in general. So, a strong course of action for the future dataset would be to collect the magnetic field data, both from heterogeneous smartphones, as well as, various brands of the same smartphone company. It will greatly help to devise intuitive solutions that can work with heterogeneous devices.

Another equally important aspect of the magnetic field datasets required for testing the positioning approaches is smartphone orientation. In real-life scenarios, users’ actions with the smartphone are complex involving various orientations. For example, navigation, call listening, phone swinging, and phone in the pockets are only a few activities that the users carry out. Magnetic field data is highly sensitive to change in orientations, and its *x*, *y*, and *z* components change substantially during such actions. It introduces large errors during the real-time positioning if the change in phone orientation is not modeled properly. To test such approaches the magnetic field dataset should include the data for various smartphones orientations which none of the publicly available datasets currently do.

In addition to smartphone heterogeneity and orientation, two important interrelated aspects that should be covered in the dataset are dynamicity and space diversity. They are co-related because traditionally large spaces tend to be more dynamic than small spaces. Large places with high mobility should be covered for the magnetic field data, such as airports, shopping malls, and train stations, etc. Similarly, most of the testing paths used for experiments are straight corridors with few turns where the user walks alongside the set path. On the other hand, real-life scenarios are complex, do not follow straight paths, and involves interruptions here and there during the walk. These scenarios should be modeled for the magnetic field datasets.

One of the publicly available datasets includes the data where the surveyor speed varies during the data collection. However, it is not enough and further data should be provided for extensive testing of the magnetic field-based positioning. Traditionally, dynamic time warping is used to compare two data sequences of varying lengths for the positioning with the magnetic field data, but such solutions are not robust. Lacking the data for varying walking speeds adds more difficulty for evaluating these systems. To check alternative solutions, the magnetic field data from more users with various walking speeds are desired. In the end, for providing the striking advantages and shortcomings of the publicly available magnetic field datasets, Table 1 summarizes the above-discussed datasets.

## 6. Conclusions

With the increase in the use of smartphones, positioning specific services experienced an escalating interest during the last few years. Precise position information of the smartphone users serves as the key element for such services. Consequently, many new technologies have emerged to assist in the positioning task for indoor and outdoor positioning. The magnetic field-based indoor positioning has been investigated as a substitute for Wi-Fi, UWB, and Bluetooth. It is well suited for indoor positioning due to its ubiquity, infrastructure independence, and magnetic sensor availability in smartphones. However, the lack of appropriate publicly available benchmark datasets makes it very difficult to evaluate the performance of magnetic field-based positioning approaches.

This study determines the most important characteristics for magnetic field datasets and divides them into basic and advanced characteristics. Two categories contain various elements of the magnetic field dataset called DUST and DOWTS. The study finds that none of the publicly available benchmarks cover these characteristics, neither basic nor advanced. So, in the future, more datasets should be gathered to investigate the important aspects of magnetic field positioning. The most important of these factors are smartphone heterogeneity, orientation, data collection in large spaces with high mobility and complex paths. The dataset that covers these characteristics would greatly help to propose and test the novel positioning approaches that use the magnetic field data.

## Figures and Tables

**Table 1:** Analysis of the available magnetic field benchmark datasets.

Ref	Basic Characteristics				Advanced Characteristics				
	Device	User	Space	Time	Dynamicity	Orientation	Walk	Trajectory	Sensor Fusion
[51]	No	No	No	No	No	No	No	Complex	Yes
[38]	Yes	No	Yes	No	Yes	No	No	Simple	Yes
[52]	No	No	No	Par	No	No	No	Complex	Yes
[53]	No	No	No	No	No	No	No	Medium	Par
[54]	Yes	Yes	No	No	No	No	Yes	Medium	Par
[55]	Yes	No	No	No	No	No	No	Simple	Par

## Data Availability

Not applicable.
